# A facile, versatile hydrogel bioink for 3D bioprinting benefits long-term subaqueous fidelity, cell viability and proliferation

**DOI:** 10.1093/rb/rbab026

**Published:** 2021-06-14

**Authors:** Hongqing Chen, Fei Fei, Xinda Li, Zhenguo Nie, Dezhi Zhou, Libiao Liu, Jing Zhang, Haitao Zhang, Zhou Fei, Tao Xu

**Affiliations:** 1 Department of Neurosurgery, Xijing Hospital, Fourth Military Medical University, Xi’an 710032, China; 2 Department of Neurosurgery, Central Theater General Hospital, Wuhan 430010, China; 3 Department of Ophthalmology, Xijing Hospital, Fourth Military Medical University, Xi’an 710032, China; 4 Biomanufacturing and Rapid Forming Technology Key Laboratory of Beijing, Department of Mechanical Engineering, Tsinghua University, Beijing 100084, China; 5 Key Laboratory for Advanced Materials Processing Technology, Ministry of Education, Department of Mechanical Engineering, Tsinghua University, Beijing 100084, China; 6 Department of Neurosurgery, Sichuan Provincial People's Hospital, University of Electronic Science and Technology of China, Chengdu 610072, China; 7 Chinese Academy of Sciences Sichuan Translational Medicine Research Hospital, Chengdu 610072, China; 8 Department of Orthopedics, Fourth medical center of PLA general hospital, Beijing 100048, China; 9 East China Institute of Digital Medical Engineering, Shangrao 334000, China; 10 Department of Precision Medicine and Healthcare, Tsinghua-Berkeley Shenzhen Institute, Shenzhen 518055, China

**Keywords:** bioink, bioprinting, subaqueous fidelity, cell viability

## Abstract

Both of the long-term fidelity and cell viability of three-dimensional (3D)-bioprinted constructs are essential to precise soft tissue repair. However, the shrinking/swelling behavior of hydrogels brings about inadequate long-term fidelity of constructs, and bioinks containing excessive polymer are detrimental to cell viability. Here, we obtained a facile hydrogel by introducing 1% aldehyde hyaluronic acid (AHA) and 0.375% *N*-carboxymethyl chitosan (CMC), two polysaccharides with strong water absorption and water retention capacity, into classic gelatin (GEL, 5%)–alginate (ALG, 1%) ink. This GEL–ALG/CMC/AHA bioink possesses weak temperature dependence due to the Schiff base linkage of CMC/AHA and electrostatic interaction of CMC/ALG. We fabricated integrated constructs through traditional printing at room temperature and *in vivo* simulation printing at 37°C. The printed cell-laden constructs can maintain subaqueous fidelity for 30 days after being reinforced by 3% calcium chloride for only 20 s. Flow cytometry results showed that the cell viability was 91.38 ± 1.55% on day 29, and the cells in the proliferation plateau at this time still maintained their dynamic renewal with a DNA replication rate of 6.06 ± 1.24%. This work provides a convenient and practical bioink option for 3D bioprinting in precise soft tissue repair.

## Introduction

Three-dimensional (3D) bioprinting technology owns the capability to manufacture a variety of living tissues with high fidelity to achieve precise repair of tissue defects, especially for hard tissue repair [1–3]. However, in soft tissue engineering, especially for deep soft tissue like the brain, blood vessel and muscle, due to the shrinking/swelling behavior of hydrogel bioinks, the lack of long-term fidelity of printed structure is still a problem to be solved in clinical transformation [4–6]. The shrinking behavior often results in incomplete filling of tissue defects and makes the internal network of the hydrogel too dense for nutrient and oxygen delivery [7, 8]. The swelling behavior results in delayed tissue repair caused by the high tension of the defect, as well as structural fragmentation caused by continuous internal mechanical loads [9, 10]. To date, most reports have attempted to achieve precise repair of soft tissues by improving bioink viscoelasticity or *in vivo* lithography bioprinting [8, 11, 12]. However, printing fidelity alone is incapable to guarantee the long-term subaqueous or *in vivo* fidelity of the constructs [13–15]. Moreover, the encapsulated cells are usually short of long-term cell viability in these constructs [14, 16, 17]. To address this hurdle, it is necessary to seek a bioink that can achieve long-term subaqueous fidelity and cell viability of biological constructs.

Gelatin (GEL) and alginate (ALG) is the most studied and mature bioink systems due to their biocompatibility, facile usability and affordability [14, 18]. Therefore, improving this bioink system is an economical and practical way to achieve the requirements above. In the GEL-ALG system, the shrinkage of constructs is usually caused by the high concentration of calcium ion crosslinker and the continuous dissolution of the hydrogel [19, 20]. The shrinking behavior might be usually counteracted by increasing GEL [21, 22]. However, excessive polymer makes the printed structures too dense for matter exchange, which is not conducive to cell viability [21, 22]. Meanwhile, the temperature responsiveness of GEL also limits its application scenarios [18, 23]. Compared with GEL, hyaluronic acid (HA) and chitosan own stronger water absorption and water retention capabilities and both are common biocompatible natural polymers [24–26]. Therefore, it is possible to achieve balanced shrinking/swelling performance by adding small amounts of these two polymers to the ALG–GEL ink. These two polysaccharides have been widely used in the form of aldehyde HA (AHA) and *N*-carboxymethyl chitosan (CMC) to prepare injectable cell-laden hydrogels based on the Schiff base linkage. Moreover, AHA and CMC are heat-insensitive below physiological temperature [23], which can improve the tolerance of printing conditions.

Based on the above, we proposed to mix the two mature systems of GEL–ALG and CMC/AHA to obtain a hydrogel bioink for the printing of subaqueous dimensional-stable constructs and maintaining cell viability. Moreover, CMC could form crosslinked networks with ALG based on electrostatic interaction [18, 27], which will promote the integrity and stability of the printed structure together with the Schiff base linkages of CMC/AHA [18, 28]. We tested the viscoelastic property, printability and shaping ability of this hydrogel under different conditions, evaluated the long-term subaqueous fidelity of the bioprinted constructs and measured the long-term cell viability and proliferation ability of the encapsulated cells through both traditional methods and flow cytometry, aiming to provide a facile and practical bioink of 3D bioprinting for precise soft tissue repair.

## Experimental section

### Synthesis of AHA

One gram of HA (Mn = 1000–1500 kDa, 1604073, Freda, China) was dissolved in 100 ml of ultrapure water, with stirring and gradually heating to 60°C. Then, the HA solution was cooled to room temperature. After wrapping the container with aluminum foil, 2.5 ml of 0.5 M (50% equivalent) aqueous sodium periodate solution was added, and the reaction was stirred at room temperature for 24 h. To remove unreacted sodium periodate, 0.25 ml of ethylene glycol was added and stirred for 2 h. The solution was dialyzed (MD34, MWCO 14000) against ultrapure water for 3 days, and the water was refreshed every 8 h. Next, The solution was lyophilized under reduced pressure (100F, SCIENTZ, China). The AHA samples were sealed and stored at 4°C for subsequent use. The molecular weight of AHA was detected by gel chromatography with glucan as standard.

### Aldehyde assay

After mixing 100 μl of 5% (w/v%) AHA solution in ultrapure water and 100 μl of 30 mM *tert-*butyl carbazate (*t*-BC) solution in 1% trichloroacetic acid, the reaction was stirred at room temperature for 24 h. Hundred microlitres of the reactant was transferred into 1 ml of 6 mM 2,4,6-trinitrobenzene sulfonic acid (TNBS) solution in 0.1 mM borate buffer (pH 8.0) and reacted with it for 1 h. The colored complex formed by TNBS and residual *t*-BC was then diluted 2 times with 0.5 M aqueous hydrochloric acid. Then, 220 μl of the diluted complex was transferred into a 96-well plate and the absorbance was measured using a microplate reader (Epoch 2, Biotek) at 334 nm. The blank was composed of aqueous 1% trichloroacetic acid and 6 mM TNBS solution in borate buffer at a volume ratio of 1:10 (also diluted 2 times by 0.5 M aqueous hydrochloric acid). The calibration curve was obtained using aqueous *t*-BC solutions with gradient concentrations (0, 2.5, 5, 10, 15, 20, 25 mM) to quantify the residual *t*-BC. The *t*-BC reacted with AHA calculated by subtracting the residual *t*-BC from the total *t*-BC was used to obtain the number of aldehyde groups. The aldehyde modification percentages of AHA were then calculated based on the number of aldehyde groups and glucuronates.

### Bioink preparation

Four percentage (w/v%) AHA solutions in phosphate-buffered saline (PBS) (Gibco) were prepared overnight at 4°C. Solutions of 1.5% (w/v%) CMC (Mn = 100 000–200 000, carboxylation degree: 83.42%, C832672, MACKLIN, China) and 10% GEL-2% ALG (w/v%) (GEL: V900863, ALG: A0682, Sigma) were prepared with PBS at 37°C. Then GEL–ALG–CMC solutions were acquired by mixing GEL–ALG and CMC solutions at a volume ratio of 2:1. AHA solutions were sterilized with a 0.22-μm filter. GEL–ALG–CMC solutions were sterilized by keeping it at 70 and 4°C for 30 min, respectively, for 3 cycles in total. All solutions were stored at 4°C after sterilization. To formulate the bioink, solutions were prewarmed to 37°C before use. AHA solutions were used to suspend the centrifuged cells. Then, GEL–ALG–CMC solutions and cell-containing AHA solutions were mixed at a volume ratio of 3:1. Briefly, two syringes containing GEL–ALG–CMC and AHA solutions were connected via a Luer taper after the air was evacuated. To avoid air bubbles during mixing, one of the plungers was gently pushed to evacuate the air in the Luer taper before tightening the interface. The bioink was finally obtained by gently pushing the two plungers alternately. The final concentration of GEL, ALG, CMC and AHA was 5%, 1%, 0.375% and 1%, respectively.

### Macroscopic gelation

To observe the hydrogel formation macroscopically, the prewarmed GEL–ALG–CMC and AHA solutions were mixed in an EP tube with a few seconds of vibrating and then kept at 37°C for 5 min. The EP tube was inverted to see if the hydrogel spread down the tube wall.

### Fourier-transform infrared spectroscopy measurement

Fourier-transform infrared spectroscopy (FT-IR) analyses were performed using a spectrometer (VERTEX 70, Bruker Daltonics, Germany). The samples were lyophilized and measured in the range of 4000–400/cm. Water vapor compensations were performed after measurements.

### Rheological analysis

Rheological testing was performed using a Haake RheoStress 1 rheometer (Thermo scientific, Germany) with a cone-plate fixture (C35/2°Ti L) at a gap of 1 mm. The temperature was set to 37°C before testing. Prewarmed solutions were loaded on the plate in sequence and mixed by a pipette for 5 s. For gelation time, the oscillation time sweep (CS, 3 Pa, 0.5 Hz) was performed to inspect the time dependence of the storage (*G*′) and loss modulus (*G*″). The Oscillation temperature ramp (CS, 3 Pa, 0.5 Hz) was performed after time sweep to test the hydrogel stability in the preset temperature range (37–10°C in 30 min, a 10-min interval at 10°C, 10–37°C in 30 min). For mechanical properties at room temperature, the temperature was reduced to 25°C in 20 min after time sweep. The oscillation frequency sweep (CD-AS, 0.01–10 Hz, 1% strain) and the oscillation amplitude sweep (CD-AS, 0.1–10 000% strain, 0.5 Hz) were performed to test the frequency dependence of *G*′ and *G*″, and the yield stress of the hydrogel, respectively. To test mechanical stability in printing, a 5-cycle deformation testing (CD-AS, 500% and 1% strain, 0.5 Hz, 200 s per cycle) was performed at 25°C to observe the instantaneous recovery property of the hydrogel.

### Printability testing

The GEL–ALG/CMC/AHA hydrogel ink was prepared without cells as described above. Both the thoroughly 25°C-cooled and the freshly 37°C-prepared hydrogel were used for printing. Different specifications of the grid structure and tubular structure were printed by a bioprinter (Livprint Norm, Medprin, China) with a 25-G nozzle (inner diameter, 0.26 mm) at a linear speed of 6 mm/s. For grid structure, the interlayer offset was 90°, and the infill rate was 30%. To simulate *in vivo* printing, a six-layer grid structure was printed on a 37°C stage. The microscopic morphology of GEL-ALG/CMC/AHA constructs was observed under a scanning electron microscope. The 10%GEL–2%ALG solutions were diluted with an equal volume of normal saline to obtain a 5%GEL–1%ALG solution and then used for printing.

### Cell culture

NIH/3T3 fibroblasts (CRL1658, ATCC) were kindly provided by the Kunming Cell Bank, Chinese Academy of Sciences (Kunming, China). Cells were cultured in high glucose Dulbecco’s Modified Eagle Medium with l-glutamine and pyruvate (HG-DMEM, 11995065) containing 10% fetal bovine serum (FBS, 10099141), 100 U/ml penicillin and 100 μg/ml streptomycin (15140122) (all from Gibco) at 37°C with 5% CO_2_. The medium was refreshed every 72 h.

### Bioprinting and culture of cell-laden constructs

Solutions were prewarmed to 37°C before use. Cells were harvested and suspended by HA solutions and then mixed with GEL-ALG-CMC solutions through two syringes for 20 s. The final concentration of cells was 1.5 × 10^6^/ml. Then, the cell-encapsulated GEL–ALG/CMC/AHA hydrogel was used to print a 6-layer grid structure with a size of 12 mm × 12 mm. After reinforced with 3% sterilized calcium chloride solutions for 20 s and washed 3 times by PBS, cell-laden constructs were transferred into a 6-well ultra-low attachment plate (Corning 3473, USA) and incubated in HG-DMEM containing 10% FBS, 100 U/ml penicillin and 100 μg/ml streptomycin at 37°C and 5% CO_2_ with the medium refreshed every 48 h. The cell-laden GEL–ALG constructs were printed as described before [29]. Briefly, harvested cells were suspended in PBS and mixed with an equal volume of GEL–ALG solution to obtain the 5% GEL–1% ALG (w/v%) bioink with the same cell concentration (1.5 × 10^6^/ml) as the GEL–ALG/CMC/AHA bioink. After printed at 10°C, cell-laden GEL–ALG constructs were crosslinked with 3% calcium chloride solutions for 2 min to achieve reinforcement and washed three times with PBS.

### Subaqueous dimensional change measurement of cell-laden constructs

The cell-laden constructs were printed and cultured as described above. The dimensions of the constructs were measured and recorded by a stereomicroscope on days 0, 1, 3, 7, 15 and 30, respectively. Formulations with different concentrations of AHA and CMC were also printed and tested.

### Live/dead assay

Live/dead viability/cytotoxicity assay kit (KGAF001, KeyGEN BioTECH, Nanjing, China) was used to test cell viability. According to the manufacturer’s instructions, both cellular imaging and flow cytometric analyses were performed. In brief, working solutions with 2 μM calcein-AM and 8 μM propidium iodide (PI) were prepared in PBS just before use. The constructs were washed three times with PBS and immersed in working solutions at room temperature for 30 min. Images were captured with an inverted fluorescence microscope (Nikon Eclipse Ti2-u, Japan). For flow cytometric analyses, stained cells in GEL–ALG/CMC/AHA constructs were isolated by dissolving hydrogels with 55 mM sodium citrate and 20 mM ethylenediamine tetraacetic acid solution and digesting cell clusters with 0.25% trypsin at 37°C for 3 min. The residual trypsin was inactivated with an equal volume of medium containing 10% FBS. Cell suspensions were further diluted with PBS and analyzed with a flow cytometer (CytoFLEX, Beckman Coulter Life Sciences). GEL–ALG constructs were treated in the same manner. Cell-free constructs were used to exclude the influence of hydrogel pieces in cell suspensions and the background of dyes. Cells cultured in the 2D environment were set as negative controls. Cells treated by 0.1% (v/v%) apoptosis inducers A and B in an apoptosis inducer kit (C0005, Beyotime, Shanghai, China) for 12 h were used as positive controls.

### Cell proliferation assay

Cell proliferation and proliferation rate assay were detected with Alamar Blue Kit (40202ES76, YEASEN, Shanghai, China) and Cell-Light ethynyl-deoxyuridine (EdU). DNA Cell Proliferation kit (containing EdU, Apollo staining buffer and Hoechst 33342; C10338-3, Ribobio, China), respectively. The initial cell number of GEL-ALG/CMC/AHA constructs and GEL-ALG constructs was both 2.5 × 10^6^. For 2D environment, the same number of cells was seeded in a six-well plate for cell culture. For the Alamar Blue assay, the working solutions were prepared by diluting Alamar Blue reagent in HG-DMEM at the volume ratio of 1:9 just before use. Cell-laden constructs were incubated with the working solutions at 37°C and 5% CO_2_ for 2 h. Hundred microlitres of the reaction solutions of each construct was transferred to a 96-well plate for absorbance measurement (570 and 600 nm). After removing the reaction solutions, the cell-laden constructs were washed twice with PBS and were continued cultured for subsequent tests. For EdU incorporation assay, 20 μM EdU solutions in the medium were used to incubate cell-laden constructs at 37°C and 5% CO_2_ for 8 h. After washing twice with PBS, the cell-laden constructs were stained with Apollo fluorescent staining buffer at room temperature for 30 min. Then the constructs were stained with Hoechst 33342 for 30 min. After that, the cell-stained constructs were washed and dissolved as described above. Cell suspensions were diluted with PBS and used to perform flow cytometric analyses. Cell-free constructs were applied to help define the cell range. Cell-laden constructs without EdU labeling were negative controls, also used for background analyses of dyes.

### Statistical analysis

The results were expressed as mean ± SD. Statistical differences between two sets of data were analyzed with two-tailed Student’s *t*-tests. Three sets of data were analyzed with one-way analysis of variance and Turkey *post hoc* tests. *P *<* *0.05 was considered statistically significant.

## Results

### Chemical characterization

**Figure 1.**
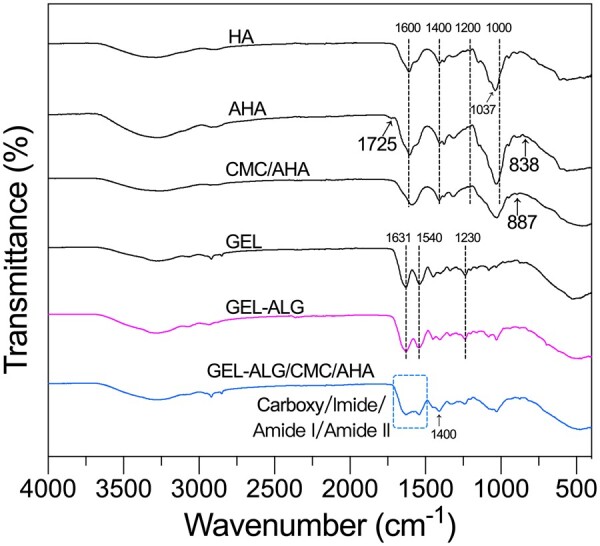
FT-IR spectra of HA, AHA, GEL, GEL–ALG, CMC/AHA and GEL–ALG/CMC/AHA hydrogels.

The actual aldehyde modification percentage of AHA was 36.44 ± 2.11%. The molecular weight of AHA was ∼14.7 kDa. The oxidation of HA by sodium periodate introduces aldehyde functions following the breaking of the C–C bond of *cis* vicinal diols in d-glucuronic acid [30]. In FT-IR analyses (Fig. 1), we observed the appearance of the symmetric stretching vibrational band (C=O) near 1725/cm [30] and out-of-plane bending vibrational band (C–H) near 838/cm [31] of aldehyde functions in AHA. The characteristic asymmetric and symmetric stretching vibration (C=O) near 1600 and 1400/cm of carboxy groups were not affected during oxidation [32]. After the mixing of CMC and AHA, the aldehyde and amino functions were crosslinked via Schiff base linkage to form the imine groups, the associated stretching vibrational band (near 1650/cm) [33] were overlapped with the vibrational bands of carboxy groups in AHA (near 1600/cm) [32] and CMC (near 1580/cm) [34]. The band near 887/cm was related to hemiacetal structures of the CMC/AHA hydrogel [35]. In the fingerprint area, the characteristic bands of the C–C, C–OH and C–O bonds in monosaccharide units of HA between 1200 and 1000/cm changed due to the alteration of the conformational freedom in the polymer chains after oxidation, especially after crosslinking [32]. For the mixture of GEL and ALG, the bands near 1631 (C=O stretch), 1540 (N–H bend coupled with C–H stretch) and 1230/cm (N–H bend) corresponded to amides I, II and III of GEL [36], respectively, while the bands related to ALG were overlapped with those of GEL. For GEL–ALG/CMC/AHA hydrogel, the vibrational bands were consistent with the CMC/AHA hydrogel and the GEL–ALG mixture, the vibration of carboxy, imine, amide I and II groups were merged into a broad band covering their respective characteristic bands.

### Gelation characteristics

**Figure 2.**
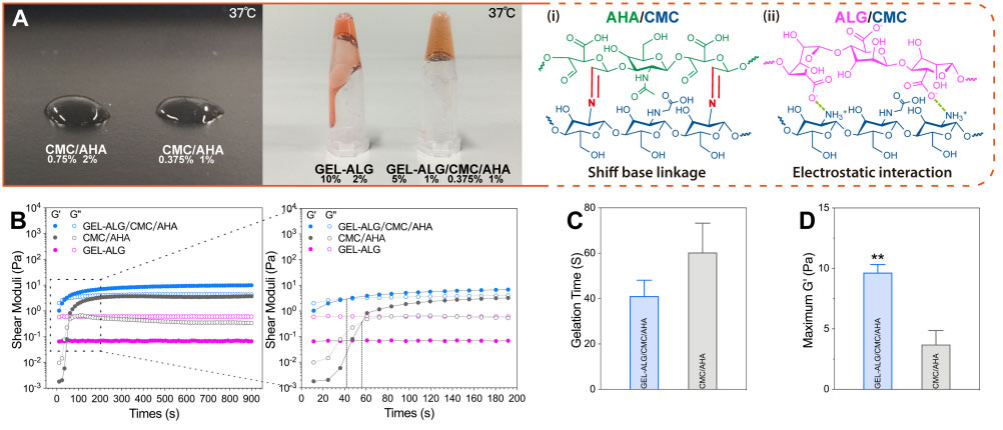
Gelation characteristics. (A) Macroscopic appearance of CMC/AHA hydrogels, GEL–ALG mixture and GEL–ALG/CMC/AHA hydrogel at 37°C and the schematic views of gelation mechanism (i and ii). (B) Gelation profile of 5%GEL–1%ALG/0.375%CMC/1%AHA, 0.375%CMC/1%AHA and 5%GEL–1%ALG. Data statistics of gelation time (*G*′ > *G*″) (C) and maximum *G*′ (D) of %5GEL–1%ALG/0.375%CMC/1%AHA and 0.375%CMC/1%AHA (*n* = 3, error bars, mean ± SD). **P <* *0.01, between different hydrogels.

Although both 0.375%CMC/1%AHA and 0. 75%CMC/2%AHA hydrogels were too soft to maintain their own shape after the EP tube was inverted. CMC/AHA hydrogels still manifested a macroscopic gel phase when located on a desktop (Fig. 2A). The GEL–ALG (10–2%, w/v%) solution was in the sol-phase at 37°C due to the temperature response of GEL (Fig. 2A). A hydrogel formed 5 min after we mixed GEL–ALG–CMC and AHA solutions at a volume ratio of 3:1 (Fig. 2A). The gelation was mainly formed by the Schiff base reaction between aldehyde functions of AHA and amino functions of CMC (Fig. 2A(i)), while the electrostatic interaction between cationic CMC and anionic ALG played a secondary role (Fig. 2A(ii)) [18, 23, 27]. When the *G*′ was higher than the *G*″, it indicated that the bioink had performed a sol-gel transition (Fig. 2B). The sol–gel transition (*G*′ = *G*″) time of GEL-ALG/CMC/AHA hydrogel was not statistically different from that of CMC/AHA (0.375%/1%, w/v%) hydrogel (Fig. 2C, 41.08 ± 7.05 vs. 60.30 ± 12.95 s, *P *=* *0.087), while the maximum *G*′ of GEL–ALG/CMC/AHA hydrogel was more than twice that of CMC/AHA (Fig. 2D, 9.65 ± 0.67 vs. 3.70 ± 1.16 Pa, *P *=* *0.016) with the effects of the above two gelations.

### Viscoelastic properties

**Figure 3.**
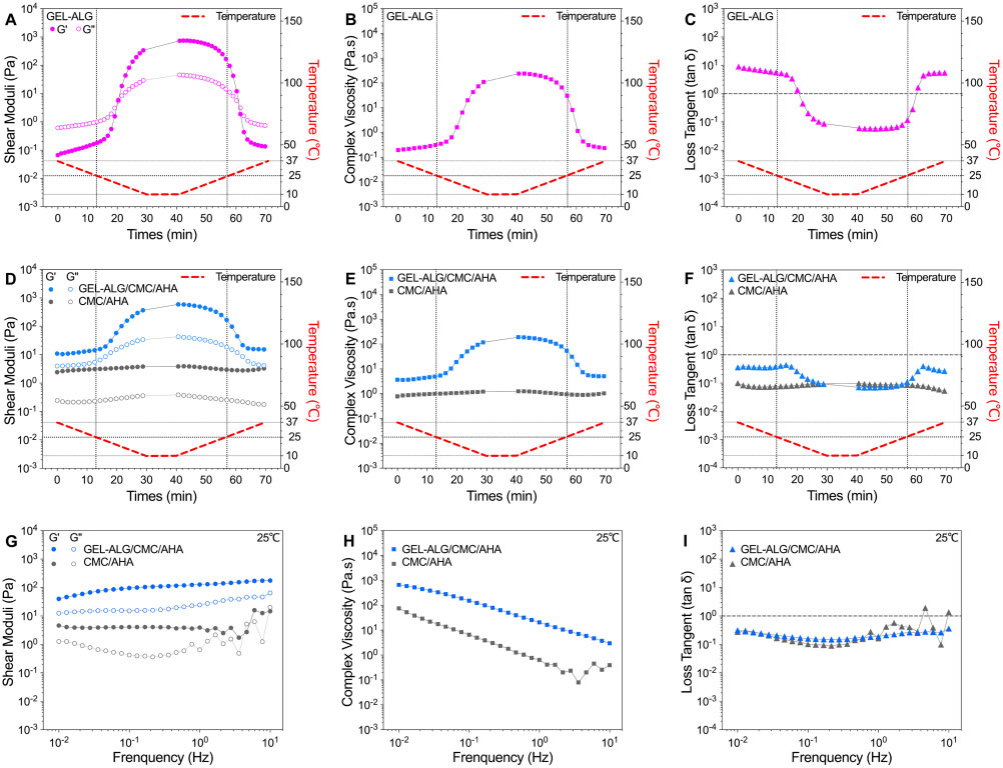
Rheological characterization of the hydrogels. Temperature dependence of shear moduli (A), |η*| (B) and tan δ (C) of 5%GEL–1%ALG. Temperature dependence of shear moduli (D), |η*| (E) and tan δ (F) of GEL–ALG/CMC/AHA and CMC/AHA hydrogels. Frequency dependence of shear moduli (G), |η*| (H) and tan δ (I) of GEL–ALG/CMC/AHA and CMC/AHA hydrogels.

Based on the thermosensitivity of GEL, the GEL–ALG (5–1%, w/v%) solution underwent a sol–gel transition with the declining temperature and returned to a sol as the temperature rose again (Fig. 3A). In such a temperature cycle, the complex viscosity (|η*|) also showed a similar change, that is increased with gelation and decreased with solation (Fig. 3B). As an indicator of viscoelastic properties, the loss tangent (tan δ) is the ratio between *G*″ and *G*′. Therefore, the temperature dependence of the tan δ of GEL–ALG was consistent with that of the shear modulus (Fig. 3C). For CMC/AHA hydrogel, the *G*′, |η*| and tan δ showed the temperature stability of crosslinking (Fig. 3D–F). For GEL–ALG/CMC/AHA hydrogel, the viscoelastic behavior was similar to that of CMC/AHA in the high-temperature range (about >23°C during cooling and >32°C during heating) and similar to that of GEL/ALG in the low-temperature range (Fig. 3D–F). The crosslinking of AHA and CMC made CMC/AHA behave in a gel state over the entire temperature range (Fig. 3D and F), while GEL changed its viscoelastic performance in different temperature ranges (Fig. 3D–F). Aiming mainly to perform printing at room temperature, we performed the oscillation frequency measurement at 25°C. The *G*′ of GEL–ALG/CMC/AHA elevated gently with increasing frequency and was more stable than that of CMC/AHA at high frequencies (Fig. 3G). The |η*| of the two samples declined with increasing frequency, both indicating the shear thinning properties (Fig. 3H) [37], of which especially suitable for extrusion printing [18, 38]. GEL–ALG/CMC/AHA can improve flow continuity during extrusion because it owns higher |η*| than that of CMC/AHA (Fig. 3H) [39]. And the value of *G*′ and |η*| in the frequency sweep were higher than that in the temperature sweep at the same frequency (Fig. 3D, E, G and H). This may be related to the hysteresis of the GEL’s temperature response [18, 23]. With Schiff base linkage, both CMC/AHA and GEL–ALG/CMC/AHA hydrogels showed elastic behavior (tan δ < 1, Fig. 3I), this lower tan δ can ensure structural integrity during printing [40]. And due to the electrostatic interaction of CMC and ALG, the tan δ of GEL–ALG/CMC/AHA hydrogel exhibited a more stable elastic property than that of CMC/AHA alone (Fig. 3I), which can ensure the stability of printed constructs [40].

### Rheological basis of shape maintenance in hydrogel printing

**Figure 4.**
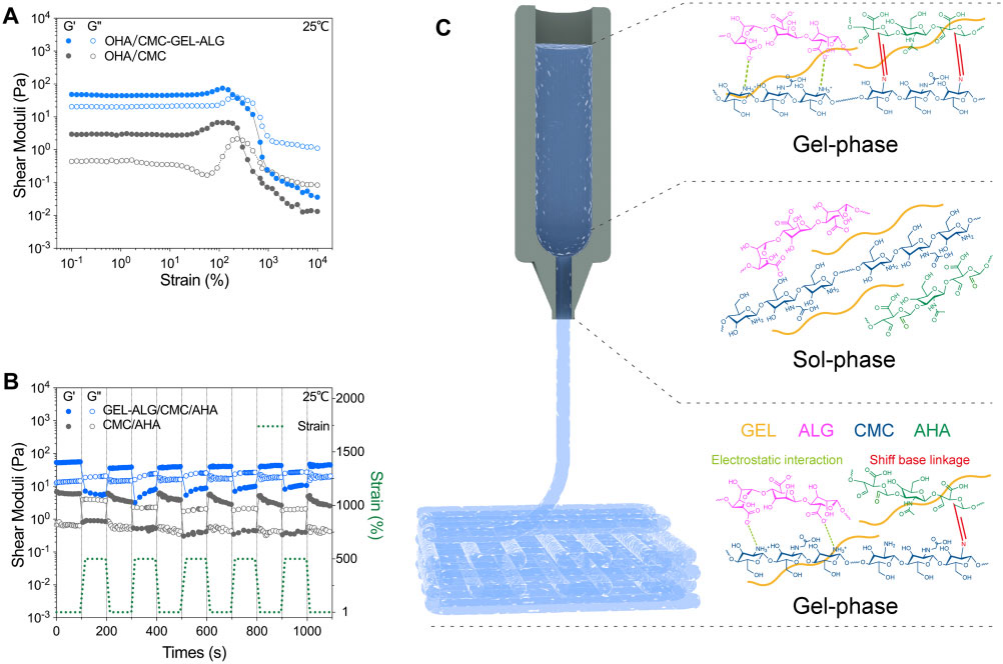
Rheological measurements of hydrogel recovery behavior. (A) Response of hydrogels to increasing strains. (B) Recovery performance of hydrogel after repeated deformations. (C) The schematic illustration of the printing feasibility of the GEL–ALG/CMC/AHA hydrogel based on the phase change of the deformation-recovery test.

The recovery ability of hydrogels after deformation is essential for maintaining the mechanical strength and shape of printed constructs [41]. The hydrogel with a higher percentage of strain recovery obtains better printing results [42]. In the oscillation amplitude sweep of CMC/AHA hydrogel, the strain at the intersection of *G*′ and *G*″ was ∼290%, indicating that the hydrogel appeared liquefied above this strain (Fig. 4A). For GEL–ALG/CMC/AHA hydrogels, due to a part of CMC participating in the weak electrostatic interaction with ALG, the minimum strain for hydrogel liquefaction is reduced to ∼235% (Fig. 4A). In the deformation-recovery test, the recovery of the stronger dynamic chemical bond in CMC/AHA after breaking is not instantaneous [18], making the *G*′ of CMC/AHA hydrogel only recover ∼50% after destructive deformation (Fig. 4B). This phenomenon is also related to the dehydration nature of the Schiff base reaction and the instability of the Schiff base linkage in an aqueous environment [43]. For GEL–ALG/CMC/AHA hydrogel, as the reversible electrostatic interaction (physical crosslinking) between CMC/ALG participated in the dissipation of mechanical energy during deformation [18, 27, 28], the *G*′ exhibited a recovery percentage of ∼80% (Fig. 4B). Therefore, the state recovery of the GEL–ALG/CMC/AHA hydrogel after deformation makes the hydrogel feasible for microextrusion bioprinting (Fig. 4C).

### Printability of hydrogel

**Figure 5.**
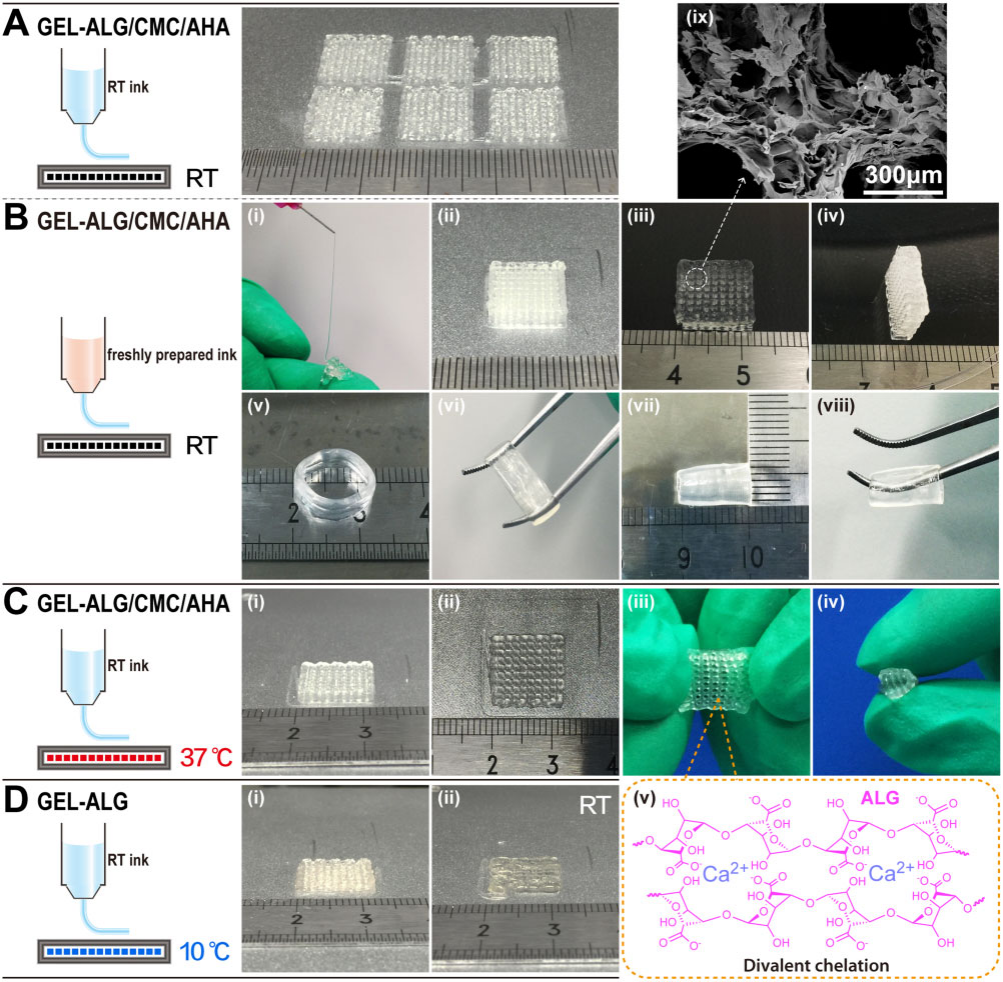
Printability of hydrogels. (A) RT GEL–ALG/CMC/AHA-printed structures (six at once). (B) Continuous extrusion of freshly formulated GEL–ALG/CMC/AHA ink (i), non-reinforced (ii) and reinforced (iii and iv) thick grid structure, reinforced tubular structures of various specifications (v–viii) and SEM micrographs of grid structure (ix) (freshly prepared GEL–ALG/CMC/AHA ink in all cases). (C) *In vivo* printing simulation (RT GEL–ALG/CMC/AHA ink and 37°C set stage), non-reinforced construct (i) and its top view (ii), construct reinforced for 2 min (iii and iv) and schematic diagram of construct reinforcement by calcium chloride (v). (D) RT GEL–ALG ink printed constructs with the 10°C set stage (i) and RT stage (ii). 24-G (inner diameter, 0.30 mm) nozzles were used for printing. No temperature intervention in the printing chamber in all cases. RT, room temperature.

When completely cooled to room temperature or used immediately after preparation, the GEL–ALG/CMC/AHA hydrogel can be continuously extruded and deposited to form various well-shaped structures (Fig. 5A and B). For *in vivo* bioprinting simulation, the simple grid structure can be printed acceptably on the 37°C stage using room temperature hydrogel (Fig. 5C). It is also important for the printed constructs to be reinforced by crosslinking with 3% calcium chloride solutions for 2 min (Fig. 5B(iii–viii) and C(iii–v)). After lyophilization, the surface of the printed filaments showed a large number of secondary micropores (Fig. 5B(ix)). This spongy microstructure was similar to that of CMC/AHA-based hydrogels [31, 44]. For traditional GEL–ALG ink, only when the platform temperature was reduced to 10°C can we obtain a well-shaped construct due to the thermosensitivity of GEL (Fig. 5D(i)), and it was impossible to print the preset shape at room temperature (Fig. 5D(ii)). The CMC/AHA hydrogel alone could not be used for printing because it was too soft to be continuously extruded into shaped filaments and effectively deposited.

### Long-term subaqueous fidelity of cell-laden constructs

**Figure 6.**
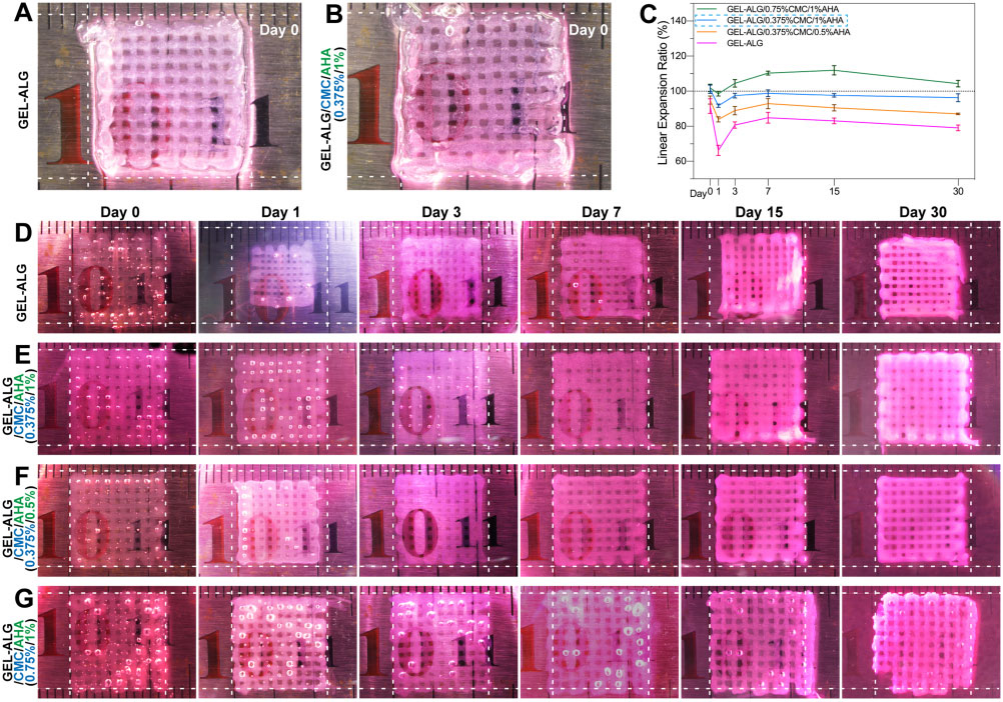
Dimensional measurements of cell-laden constructs during 30 days of culture. Photographs of freshly crosslinked cell-laden GEL–ALG (A, crosslinked by 3% calcium chloride for 2 min) and GEL–ALG/0.375%CMC/1%AHA (B, crosslinked by 3% calcium chloride for 20 s) constructs. (C) Dimensional change profile of cell-laden constructs (*n* = 3, error bars, mean ± SD). Subaqueous dimensional images of cell-laden GEL–ALG (D), GEL–ALG/0.375%CMC/1%AHA (E), GEL–ALG/0.375%CMC/0.5%AHA (F) and GEL–ALG/0.75%CMC/1%AHA (G) constructs.

We printed six-layer grid constructs with a size of 12 mm × 12 mm using the hydrogel ink with the NIH/3T3 cell concentration of 1.5 × 10^6^/ml. The GEL–ALG constructs were crosslinked with 3% calcium chloride for 2 min as our previous reports (Fig. 6A) [45]. However, when the GEL–ALG/CMC/AHA constructs were crosslinked for the same amount of time, the constructs were too rigid for cell proliferation. An optimal reinforcement was obtained about only 20 s after the calcium chloride solutions were simply spayed on the constructs (Fig. 6B), which was the minimum requirements for constructs could be separated from printing platform without shape destruction. To observe and measure the same sample continuously, we defined the ratio of the side length (SL_t_) of the grid structure to the preset side length (SL_0_) at different time points as the linear expansion ratio (LER = SL_t_ × 100%/SL_0_) instead of the traditional weight ratio for swelling behavior evaluation. For GEL–ALG bioink, the crosslinking of ALG and calcium chloride caused the hydrogel to shrink [46–48]. However, the swelling behavior of GEL (5%) in this formulation could not offset the shrinkage of constructs effectively (Fig. 6C and D). The cell-laden GEL–ALG constructs showed the most obvious shrinking behavior during the whole incubation process, the LER (66%) was the lowest when constructs were incubated for 24 h (Fig. 6C and D). Then, the constructs began to swell and shrank again due to the degradation of crosslinked networks and dissolution of GEL after day 7 (Fig. 6C and D). For cell-laden constructs printed with GEL–ALG/0.375%CMC/1%AHA hydrogel bioink, its LER was closest to 100% during the 30-day incubation due to the strong water retention capacity of HA and chitosan. Similarly, the structure also shrank due to the degradation of crosslinked networks after 7 days (Fig. 6B, C and E). When we decreased the concentration of AHA to 0.5%, the constructs could not maintain the original size (Fig. 6C and F). And when the concentration of CMC in the bioink was increased to 0.75%, the excessive water absorption and retention effect caused the constructs to exhibit obvious swelling behavior from day 3, although the constructs initially maintained its dimensional appropriately (Fig. 6C and F). In general, the GEL–ALG/0.375%CMC/1%AHA hydrogel bioink manifested a balanced water absorption and retention capacity, making the related cell-laden constructs obtained an optimal long-term subaqueous dimensional stability.

### Cell viability

**Figure 7.**
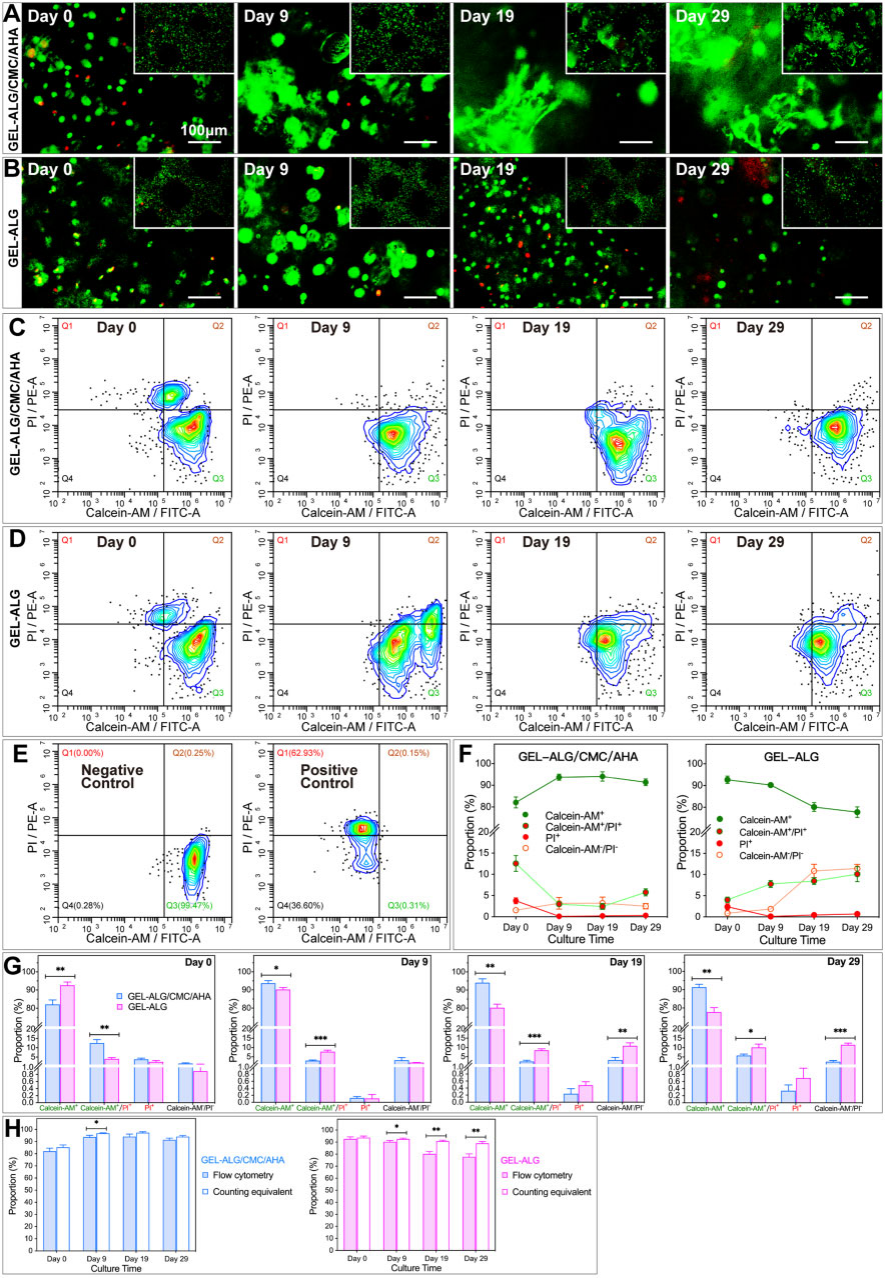
Cell viability analyses. Micrographs of live (green, calcein-AM)/dead (red, PI) stained cells in GEL–ALG/CMC/AHA (A) and GEL–ALG (B) constructs. Flow cytometry contour plots of cell viability in GEL–ALG/CMC/AHA (C) and GEL–ALG (D) constructs. (E) Cytometry plots of negative control and positive control for cell viability assay. Trends profile of the proportion of cells in different states (F) and data statistics of the distribution of cells in different states at respective time points (G) (*n* = 3, error bars, mean ± SD), based on flow cytometry data. (H) Comparison of cell viability between flow cytometry and analog counting methods (*n* = 3, error bars, mean ± SD). *P <* *0.05, **P <* *0.01, ***P <* *0.001, between cells with the same state in different constructs (G) and between cell viability under different evaluation methods (H) at each time point.

In the constructs just printed, some cells were not stained because they were located deep, while in the stained cells, the shearing force in printing caused death or membrane damage of a small part of the cells, which were stained by PI or dual stained by calcein-AM/PI, respectively(Fig. 7A– D). We observed this phenomenon by both the fluorescence images and the flow cytometry plots (Fig. 7A–D). On day 0, the proportion of calcein-AM^+^ cells in GEL–ALG/CMC/AHA (82.10 ± 2.53%) was lower than that in GEL–ALG (92.65 ± 1.73%), while the proportion of calcein-AM^+^/PI^+^ cells in this bioink (12.54 ± 1.89%) was higher than that in GEL-ALG (4.05 ± 0.65%, Fig. 7A– G). For the proportion of PI^+^ cells and unstained cells, there was no difference between these two bioinks at this time (Fig. 7G). The above distribution of cell survival status just after printing (day 0) was due to the damage of the cell membrane caused by the additional pressure that broke the covalent bonds of CMC/AHA when GEL–ALG/CMC/AHA hydrogel bioink was extruded. As the culturing continued, the proportion of calcein-AM^+^ cells in GEL–ALG/CMC/AHA constructs showed an increasing trend, while in GEL–ALG constructs it exhibited a contrary tendency (Fig. 7F). And the proportion of the calcein-AM^+^ cells in GEL–ALG/CMC/AHA was higher than in GEL–ALG on day 9 (93.71 ± 1.46% vs. 90.24 ± 1.06%), day 19 (94.08 ± 2.15% vs. 80.16 ± 2.14%) and day 29 (91.38 ± 1.55% vs. 77.75 ± 2.46%) (Fig. 7A–G). Moreover, on days 19 and 29, cells in GEL–ALG/CMC/AHA were found to become a revealing display spreading morphology (Fig. 7A). This ideal morphological basis for cell proliferation confirmed the performance of cell viability [49]. And in GEL–ALG, cells were always spherical, indicating lower cell viability (Fig. 7B) [49]. PI^+^ cells accounted for <1% of cells in both constructs from days 9 to 29 (Fig. 6G). Therefore, the decrease in the proportion of live cells (calcein-AM^+^) was not simply caused by the increase in dead cells (PI^+^). The proportion of calcein-AM^+^/PI^+^ cells in GEL–ALG/CMC/AHA decreased significantly on day 9 (2.98 ± 0.35%), day 19 (2.43 ± 0.69%) and day 29 (5.79 ± 0.82%) compared with that on day 0, while the proportion increased gradually in GEL–ALG and was at a higher level from days 0 to 29 (4.05 ± 0.65%, 7.77 ± 0.84%, 8.49 ± 0.80% and 10.10 ± 1.77% on days 0, 9, 19 and 29, respectively, Fig. 6F and G). The proportion of the unstained (calcein-AM^−^/PI^−^) cells in GEL–ALG/CMC/AHA constructs was <5% throughout the culture, while the proportion in GEL–ALG increased to >10% on days 19 and 29 (Fig. 6F and G). It is indicated that with the AHA and CMC, the balanced water retention capacity of GEL–ALG/CMC/AHA bioink not only made the size of the construct more stable but also ensured an appropriate matter exchange during culture, which led to a better cell viability.

In addition, we used flow cytometry data to calculate the counting equivalent of cell viability by simulating a microscope counting method [(calcein-AM^+^ + calcein-AM^+^/PI^+^) × 100%/(calcein-AM^+^ + calcein-AM^+^/PI^+^ × 2 + PI^+^)]. This analog counting value was affected by the unstained cells and/or calcein-AM^+^/PI^+^ cells. When these two data were large, the counting equivalent of cell viability in GEL–ALG was >90%, which was consistent with previous reports and was higher than that of flow cytometry analyses on days 9, 19 and 29 (Fig. 6G and H). However, the cell viability evaluated by these two methods in GEL–ALG/CMC/AHA constructs was only different on day 9 due to the lower proportion of unstained cells and calcein-AM^+^/PI^+^ cells (Fig. 6H).

### Cell proliferation

**Figure 8.**
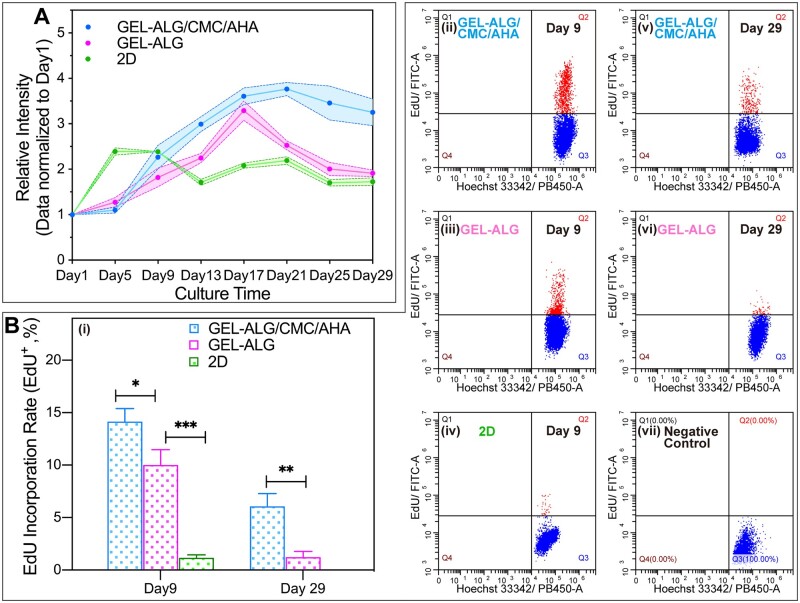
Evaluation of cell proliferation. (A) Cell proliferation profile evaluated by Alamar Blue assay (*n* = 3, error bars, mean ± SD). (B) DNA replication ability in different culture conditions evaluated by EdU incorporation assay on day 9 and 29 (i) (*n* = 3, error bars, mean ± SD), flow cytometry scatter plots of DNA replication (ii–vi) and negative controls (vii). *P <* *0.05, **P <* *0.01, ***P <* *0.001, between different culture conditions at each time point.

In the Alamar Blue assay, both of the cells in GEL–ALG/CMC/AHA and GEL–ALG constructs proliferated significantly from day 5 and reached a peak on day 17 (Fig. 8A). The cells proliferated to 3.61 ± 0.19-fold in GEL–ALG/CMC/AHA and 3.29 ± 0.22-fold in GEL–ALG (Fig. 8A). The difference was that the cell growth in GEL–ALG/CMC/AHA was still in a plateau stage from days 17 to 29, while the cell growth in GEL–ALG showed a decreasing trend after day 17 (Fig. 8A). The same number of cells was also cultured in a 2D environment; the cells reached the growth plateau stage with 2.39 ± 0.08-fold on day 5 due to insufficient growth space and then began to decrease and fluctuate after day 9 (Fig. 8A). In addition, EdU incorporation assay can detect the cell proliferation more sensitively [50–52], was also performed on days 9 and 29 to verify our results. The results were consistent with those of the Alamar Blue assay as the above. On day 9, the EdU incorporation rate of cells in GEL–ALG/CMC/AHA (14.16 ± 1.23%) was higher than that in GEL–ALG (10.01 ± 1.45%), while the EdU incorporation rate of cells in 2D was only 1.17 ± 0.29% (Fig. 8B(i)–(iv)). On day 29, although the cell proliferation in GEL–ALG/CMC/AHA constructs was in the plateau phase in Alamar Blue assay (Fig. 8A), there was still a 6.06 ± 1.24% EdU incorporation rate (Fig. 8B(i) and (v)), indicating that the cell proliferation was still going on at this time. For GEL–ALG constructs, the EdU incorporate rate of cells was only 1.23 ± 0.55% (Fig. 8B(i) and (vi)), indicating that almost no cell proliferation occurred at this time.

## Discussion

The convenience and practicability of 3D bioprinting are not only requirements but also challenges in laboratory research and clinical applications [41]. The simple printing process and tolerant shaping conditions could shorten the time of the cells leaving the cultural environment within an acceptable range to avoid excessive loss of cell viability [18, 41]. The long-term fidelity of the constructs might ensure the consistency of cell growth space and the matching accuracy of tissue repair. Based on the above considerations, we carried out this work. In addition, the raw materials we used are all currently affordable materials with required biocompatibility and biofunctionality [18]. It is more valuable to use these biological materials to further improve the convenience and practicality of 3D bioprinting.

Printability is an important feature that distinguishes hydrogel inks from ordinary hydrogels [18, 53]. In microextrusion bioprinting, the dynamic crosslinking mechanism is one of the common ways to realize the printability of hydrogels [18, 41, 53]. The gelation of GEL–ALG/CMC/AHA is based on the Schiff base linkages of CMC/AHA and the electrostatic interaction of CMC/ALG (Fig. 2A). The increase in concentration and the dual crosslinked network bring more molecular entanglement [23, 28], thereby increasing the viscosity of the GEL–ALG/CMC/AHA hydrogel (Fig. 3B, E and H). High viscosity can facilitate extrusion uniformity, shape maintenance and mechanical stability, especially for the printing of higher or more complex constructs [28, 41]. The use of GEL is not only for its good biocompatibility and biological activity but also for its considerable viscosity adjustment at lower temperatures [18, 23]. The reported ideal printable hydrogel requires a *G*′ of 30–150 Pa to achieve a self-supporting effect [54, 55]. In rheological tests, the *G*′ of the hydrogel was ∼50 Pa when it was completely cooled to 25°C, and the *G*′ recovered to ∼80% after deformation, ensuring the self-supporting of the constructs during printing (Fig. 4). Based on the higher viscosity and proper *G*′, we printed out various specifications of constructs using the GEL–ALG/CMC/AHA hydrogel at room temperature, which intuitively verified the printability of this hydrogel (Fig. 5A–C). In actual printing, due to the temperature responsiveness of GEL to natural cooling during the printing process, the hydrogel can be printed regardless of whether it waited for cooling, which greatly facilitates printing.

The hysteresis of the temperature response of GEL [18, 23] kept the *G*′ of the hydrogel in the self-supporting range for a long time during the heating process (Fig. 3D). This allowed us to use room-temperature hydrogels to fabricate simple models on a 37°C stage for *in vivo* printing simulation. For *in vivo* bioprinting, temperature-independent bioinks and short-time crosslinking are often required to facilitate superficial tissue repair or minimally invasive repair of deep tissues [11, 56]. Although nanomaterials can significantly reduce the temperature dependence of bioinks, improve printability and enhance mechanical strength, there is still a lack of consistent positive conclusions about their biological effects [28, 42]. Other biocompatible viscosity modifiers, such as agarose, have a higher response temperature than GEL [23], making it possible to achieve a more stable bioink at physiological temperature. However, due to its higher viscosity, this type of bioink was usually printed at a lower concentration (1.5%) at 15°C, and the immediate cell viability after printing was only ∼75%, while the cell proliferation only lasted for 9–11 days [57, 58]. Therefore, the weak temperature dependence and short time reinforcement requirement of the GEL–ALG/CMC/AHA hydrogel provides a more practical option for *in vivo* bioprinting.

For the reinforcement of printed samples, due to the weak water solubility of GEL, traditional GEL–ALG constructs need to be crosslinked with 3% calcium chloride for 2 min to obtain the desired strength and durability [18]. In the GEL–ALG/CMC/AHA constructs, ALG is more cline to form stable divalent chelation-induced gelation with calcium ions because of its special conformation, although the carboxyl group in CMC can also react with calcium ions [27, 59]. Due to the instability of CMC/AHA in an aqueous condition [44], calcium chloride is easier to react with deeper ALG. Therefore, it only took 20 s for the sprayed 3% calcium chloride solution to crosslink with ALG appropriately, making post-printing reinforcement more convenient. In terms of microscopic morphology, the shrinking behavior caused by the crosslinking between ALG and calcium chloride often results in dense networks [14, 60], which will adversely affect the viability of deep cells [60]. Therefore, water-soluble substances can be used as a pore-forming agent to make microstructures looser for better cell viability [60]. The use of CMC/AHA in GEL–ALG/CMC/AHA made the printing filament obtain a loose spongy microstructure (Fig. 5B(ix)), providing a better structural basis for cell viability and proliferation. Although the influence of calcium chloride reinforcement time on cells in these two types of constructs was not excluded, GEL–ALG/CMC/AHA is more beneficial than GEL–ALG at long-term cell viability maintenance in terms of practicality.

Compared with the hydrogel alone, the addition of cells can more realistically evaluate the swelling/shrinking behavior of the biological constructs *in vitro*. Due to the shrinkage behavior of ALG and calcium chloride crosslinking [46–48], all the cell-laden constructs shrank to varying degrees after 24 h of culture (Fig. 6). In the subsequent cultivation, the limited water absorption and water retention capacity of GEL [24] failed to restore GEL–ALG to its original size (Fig. 6C and D). Although the size can be restored by further adding GEL, too much GEL will make the construct denser, which will adversely affect cell extension, migration, viability and proliferation [21, 22]. AHA and CMC, especially AHA, have very strong water absorption and water retention capabilities [25, 26]. This allowed us to balance the swelling/shrinking behavior of the construct with fewer materials (1% AHA and 0.375% CMC), providing a good foundation for high cell viability and proliferation [21, 22]. When the water-absorbing component was further increased, the cell-laden constructs showed swelling behavior and eventually broke (Fig. 6G). This is because the crosslinked network of the hydrogel was destroyed by the continual mechanical load caused by the swelling state [9, 10].

Cell viability is the basic requirement for cell proliferation [23, 41]. Unlike the 2D environment where cells have equal opportunities to exchange nutrients and metabolites, 3D hydrogels with insufficient micropores and/or permeability not only make it difficult for deep cells to obtain nutrients but also cause local metabolic waste accumulation [22, 61]. In fact, the advantage of 3D bioprinting is that the controllable primary macropores can ensure the matter exchange of each filament [22]. In our study, although the high viscosity of GEL–ALG/CMC/AHA resulted in lower cell viability on day 0, the abundant micropores of printed filaments made it easier to retain water and realize deep matter exchange, thus ensuring better long-term cell viability (Fig. 7A, C, F and G). In the late stage of culture, the significant reduction in live cells in the GEL–ALG construct was accompanied by an increase in dual stained and unstained cells, rather than an increase in dead cells (Fig. 7B, D, F and G). Considering the possibility of positive dual staining caused by mechanical damage to the cell membrane was excluded by performing live/dead staining before dissolving the constructs, these dual stained cells might already be in the middle and/or late stages of apoptosis, while unstained cells indicated a decrease in the permeability of the constructs. It is supposed that this is due to the obstruction of the smaller secondary micropores of GEL–ALG caused by the proliferation of cells located in the superficial position of the printed filaments in the late culture stage [46].

High molecular weight HA (>1000 kDa) participates in maintaining cell viability by simulating the extracellular matrix together with other components [62], while low molecular weight HA (10–250 kDa) and HA oligosaccharides (<10 kDa) can directly stimulate cell proliferation through CD44 receptors [63, 64]. In this study, the molecular weight of HA was reduced to 14.7 kDa after oxidation. However, the main purpose of this work is to verify the universality of a new bioink, not focusing on the construction of a specific tissue. Therefore, the CD44-negative NIH/3T3 cell line was selected. CD44 exists in a variety of cells like cancer stem cells (glioma cells [65], lung cancer cells [66], breast cancer stem cells [67], liver cancer cells [68], gastric cancer cells [69]), normal stem/progenitor cells (embryonic stem cells [70], neural stem cells [71], intestinal stem cells [72]), epithelial cells [73], vascular smooth muscle cells [74] and endothelial cells [75, 76], mediating the HA regulation of cell adhesion, proliferation, differentiation, migration and metabolism. Therefore, this facile and affordable bioink has great potential in establishing such vascularized tumor models and tissue repair through 3D bioprinting in the future.

The cells in GEL–ALG and GEL–ALG/CMC/AHA constructs formed small cell clusters in logarithmic growth phase (Figs. 7A and B and 8A). The proliferation of anchorage-dependent cells also relies on balanced adhesion to maintain optimal cell morphology, and rounded cells indicate insufficient proliferation or even apoptosis [49]. Due to the insufficient internal network of GEL–ALG, the cells were always in a spherical shape (Fig. 7B), indicating that these cells began to apoptosis after undergoing adaptive proliferation (Fig. 7D, F and G). Although similar to the other reports [77, 78], the cell proliferation in GEL–ALG in this work was not as good as that of tumor stem cells in this ink in our previous study (Fig. 8A) [45], the reason of which was that ordinary cell lines do not have the capabilities of enrichment and aggressive growth like cancer stem cells. In GEL–ALG/CMC/AHA, because the extracellular matrix-like components formed more biocompatible supramolecular crosslinked networks, the cells gradually expanded from a round shape during proliferation (Fig. 7A). However, the cells in GEL–ALG/CMC/AHA showed uneven growth (Fig. 7A), which we assume should be caused by the difference in nutrient acquisition at different locations of the microfilaments. For cell proliferation, if only the proliferation fold on day 17 was considered, the ∼3.61-fold proliferation of cells in GEL–ALG/CMC/AHA did not bring huge cell proliferation benefits compared to the ∼3.29-fold in GEL–ALG. However, the advantage of GEL–ALG/CMC/AHA is to ensure long-term dynamic proliferation by maintaining long-term high cell viability (Figs. 7 and 8). In flow cytometry analysis, the EdU incorporation rate of cells in GEL–ALG on day 29 was only 1.23 ± 0.55%, which was equivalent to the EdU incorporation rate of 1.17 ± 0.29% for cells in the apoptotic state after the plateau on day 9 in the 2D environment (Fig. 8B). Live/dead staining at this time also confirmed the apoptotic state of the cells (Fig. 7D, F and G). In GEL–ALG/CMC/AHA, the cells still had an EdU incorporation rate of 6.06 ± 1.24% on day 29 (Fig. 8(v)), indicating that the cells were in a dynamic balance between proliferation and apoptosis at this time, which was supported by the long-term high cell viability (Fig. 7A, C, F and G). This dynamic renewal mode similar to cells in living organisms indicates that the new bioink can better simulate the extracellular microenvironment.

The cell viability of the traditional counting method included dual stained cells, and the higher the proportion of such cells, the higher the cell viability value calculated. More positive dual staining indicates an increase in cell membrane permeability, which can be used to evaluate the damage to the cell membrane caused by the shearing force during printing. In the late stage of cell culture, this phenomenon predicts cell apoptosis. In our research, there was no difference between the analog counting method and flow cytometry in evaluating cell viability in both bioinks immediately after printing. However, in the late stage of culture, due to the high proportion of dual stained cells and unstained cells, the counting method overestimated the cell viability in GEL–ALG, while the cell viability assessed by flow cytometry matched the cell proliferation state more closely at this time (Figs. 7F and 8). Although there was mostly no difference between the cell viability assessed by these two methods in GEL–ALG/CMC/AHA, we recommend that when evaluating the long-term viability of cells in an unknown bioink, or when evaluating the *in vitro* culture time required before implantation of cell-laden constructs, flow cytometry can be used to assess the cell viability status more accurately. In terms of cell proliferation, flow cytometry is not inconsistent with classical methods. Therefore, flow cytometry can be used to corroborate with traditional methods.

## Conclusion

From this work, we proposed a facile GEL–ALG/CMC/AHA hydrogel bioink by simply combining two mature and affordable systems, GEL–ALG and CMC/AHA. The Schiff base linkages of CMC/AHA and the electrostatic interaction of CMC/ALG contributed to the gel phase. The addition of CMC/AHA weakened the temperature sensitivity of ALG–GEL, thereby increasing the latitude of printing conditions, making the hydrogel capable of both *in vitro* printing and simple *in vivo* simulation printing. The post-printing crosslinking time of only 20 s also further improved the convenience and practicality of the ink. The printed cell-laden constructs maintained subaqueous fidelity for 30 days. Flow cytometry showed that the cell viability was 91.38 ± 1.55% on day 29, and the cells in the proliferation plateau at this time exhibited a 6.06 ± 1.24% DNA replication rate, indicating a dynamic renewal of cells. We believe that versatile hydrogel bioink can facilitate the precise repair of soft tissues. Precise repair of specific soft tissues, especially deep soft tissues, can be implemented in the future applying this hydrogel bioink, to promote the application of 3D bioprinting in precision medicine of deep soft tissue engineering.
